# Effect of Thermal Aging on the Mechanical Properties of SAC305

**DOI:** 10.3390/ma15082816

**Published:** 2022-04-12

**Authors:** Khozima Hamasha, Mohammad M. Hamasha, Sa’d Hamasha

**Affiliations:** 1 Department of Basic Scientific Sciences, Al-Huson University College, Al-Balqa Applied University, Al-Salt 19117, Jordan; khamasha@bau.edu.jo; 2 Department of Industrial Engineering, Faculty of Engineering, The Hashemite University, P.O. Box 330127, Zarqa 13133, Jordan; 3 Department of Industrial and Systems Engineering, Auburn University, Auburn, AL 36849, USA; smh0083@auburn.edu

**Keywords:** thermal aging, SAC305, mechanical properties, solder joint, Cu6Sn5, Ag3Sn, β-Sn

## Abstract

Many electronic products are subjected to heat for long periods, depending on their operations. Thus, it is expected that the physical and mechanical properties of electronic elements, including the soldering joints, will be affected. In this study, the impact of thermal aging time and temperature on the microstructure and mechanical properties of 96.5Sn–3.0Ag–0.5Cu (SAC305) was investigated. The samples used were SAC305 solder balls attached to copper pads. The research began by examining the microstructure of the aged samples at 150 °C for 100 and 1000 h. Then, this was compared to the microstructure of the same samples without thermal aging. Then, five groups of 10 samples were prepared from a shear stress–shear stain experiment. The first group was as produced, the second group was aged for 2 h, the third group was aged for 10 h, the fourth group was aged for 100 h, and the fifth group was aged for 1000 h. All groups were aged at a temperature of 150 °C. An Instron testing machine was used to plot a shear stress–shear stain curve until the ball was completely sheared off the pad. The mechanical properties, including the ultimate shear strength, the ultimate energy used to shear the ball, and the total energy used to shear the ball at all thermal aging times were then estimated. The results of this study indicated the formation of a layer of Cu6Sn5 over the copper pad, which thickened with thermal aging time. Furthermore, the ultimate and total shear strengths decreased with thermal aging time. The same procedure was repeated to assess the ultimate shear strength at 100 °C. The decrease in ultimate shear strength was more severe with increasing thermal aging temperature.

## 1. Introduction

Solder joint reliability is a critical concern because they provide mechanical support and electrical and thermal connections between the various components in microelectronic packages. Before fabricating solder joints, various factors must be addressed during the soldering process. Solder paste selection, temperature difficulties, reflow profiles, and component size variations are all considered.

Electronic packaging is essential in the electronic manufacturing industry because it provides both electronic and mechanical connections, as well as protection for electronic components [1]. Electronic devices are gradually becoming more miniaturized and multifunctional as the electronics industry rapidly develops [2]. As a result, to meet people’s needs, the electronic packaging industry is moving toward denser, more efficient, and more integrated products.

As electronics continue to get smaller and more complex, maximum mechanical and thermal loads must not exceed the solder joints’ thermal resistance. In addition, lead-free solder materials are currently required because traditional tin–lead soldering cannot meet the environmental and health requirements of the electronics industry [3]. Therefore, a very large number of studies have been performed to develop lead-free alloys [4,5,6,7].

One of the major challenges arising from packaging materials and process modifications during the transition to lead-free materials is the reliability of lead-free solder joints. The abilities of packaging systems are used to keep the operation of the electronic components from degrading and, thus, to increase the reliability of the entire electronic package. Solder joints can also serve as an electrical connection between the board and the components, as well as a heat dissipation outlet in the package [8]. However, solder joints are the weakest and most likely to fail in electronic packages. Temperature [9], vibration [10], tin whisker [11], and electromigration [12] are only a few of the extrinsic causes of solder connection failure. 

Sn–Ag–Cu alloys have been identified as the most promising lead-free solder candidate among many alloy systems due to their relatively low melting temperature, good mechanical qualities, and good compatibility with other components [13]. Sn–Ag–Cu alloys are commonly used as solder balls and pastes in the microelectronic packaging industry. However, no “drop-in” alternative is suited to all situations. SAC305 (96.5Sn–3.0Ag–0.5Cu) is described as one of the most commonly used alloys in solder joints [14]. 

Environmental exposures such as thermal aging and thermal cycling dramatically modify the SAC lead-free solder microstructure [15]. In consequence, SAC solder joints tend to fail. Previous studies showed a considerable reduction in the strength and modulus of elasticity and other mechanical properties due to thermal aging [16]. Lee et al. [17] reported a degradation in the ball shear strength of SAC alloys due to thermal aging. Furthermore, Zhang et al. [18] showed that the creep rate is also affected by thermal aging. Additionally, Lall et al. [19] concluded that thermal aging significantly affects the performance of SAC alloys given high strain rates.

The microstructure evolution during the thermal cycling of Sn–Ag-based solder interconnects was examined by Chen et al. [20]. They observed the recrystallization and the segregation of Ag3Sn intermetallic compound particles in those regions. Scanning electron microscopy (SEM) and optical microscopy (OM) were utilized to observe the influence of mechanical and thermal loadings on SAC alloys by Matin et al. [21]. The study discovered voids and microcracks in the eutectic regions. Propagated damage caused by the grain boundaries of thermal loads was also noticed. 

Hu et al. [22] studied the effect of thermal aging on the microstructure of SAC305 and its shear strength. However, the shape of the solder used was flat, not spherical. The flat shape does not the mimic ball-shaped solder joints used in real applications. Therefore, the shear strength, in particular, is far from the true application results. Power et al. [23] and Lall et al. [24] applied a thermal aging experiment for a long period of time, estimated in days, and examined the microstructure. Power et al. [23] applied thermal aging at 125 °C for 30 and 56 days, while Lall et al. [24] conducted a similar experiment but at a relatively low temperature of 100 °C for a longer period of 100 days. Long periods bring the material to a stable state in the microstructure, which does not show gradual variation during the thermal aging process. Al Athamneh [25] and Chowdhury et al. [26] studied the effect of heat aging on the fatigue life of SAC305. They designed fatigue cycling experiments for this purpose. Zhao et al. [27] applied thermal aging and then thermal cycling on the same samples, and they subsequently analyzed the microstructure. Hasnine et al. [28] investigated the effect of aging on the hardness of SAC305.

Although several researchers have investigated the microstructure of SAC305 after long-term thermal aging, the investigation of short-term thermal aging (less than 40 h) has not yet been covered. For example, the authors of [22] started to investigate the microstructure at 48 h. Furthermore, mechanical properties, such as stress–strain behavior, ultimate shear strength, and toughness (or energy expended), have not been addressed in previous research. Therefore, the research in this paper was designed to cover this unstudied area, as detailed in the next section.

## 2. Experimental Procedure

The experiment was conducted in three phases, sample preparation, thermal aging, and mechanical property quantification. The phases are explained in the three subsections below. In applications, shear stress may be applied to solder joints due to thermal expansion of the component connected to the solder joints.

### 2.1. Sample Preparation

**Figure 1:**
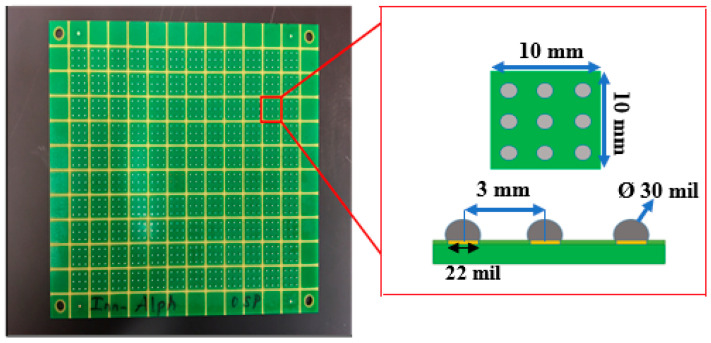
Test vehicle design.

**Figure 2:**
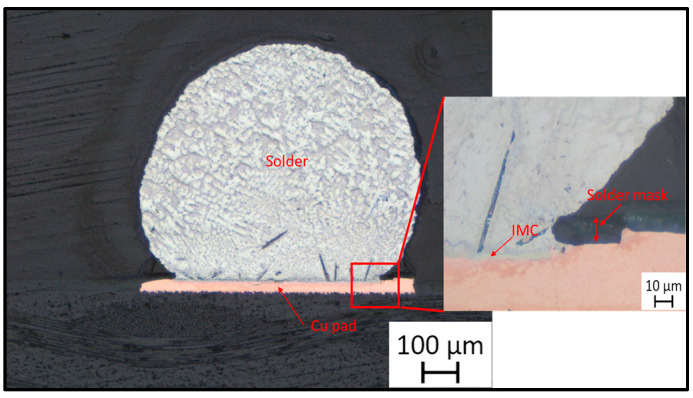
Cross-section of a solder joint after soldering to a copper plate.

In the present work, SAC305 solder alloys have been used as the test material. Solder balls 0.75 mm in diameter were placed directly onto 0.55 mm diameter copper pads on ideal printed circuit boards (PCB). Then, the boards were placed at a temperature of 245 °C for 45 s to remelt the balls, thus soldering the copper pads. Each test board contained 120 squares, each square was 10 mm × 10 mm in dimension, and each square included nine solder joints (spheres). The test board and solder joints are shown in Figure 1. Moreover, the cross-section of a solder joint is shown in Figure 2. 

### 2.2. Thermal Aging

After 10 boards were prepared by soldering each copper pad with a solder sphere, the boards were ready for a thermal aging experiment. Two boards were left as they were, and eight were subjected to thermal aging for different aging times and at different aging temperatures, as explained in Table 1.

### 2.3. Shear Strength Test

**Figure 3:**
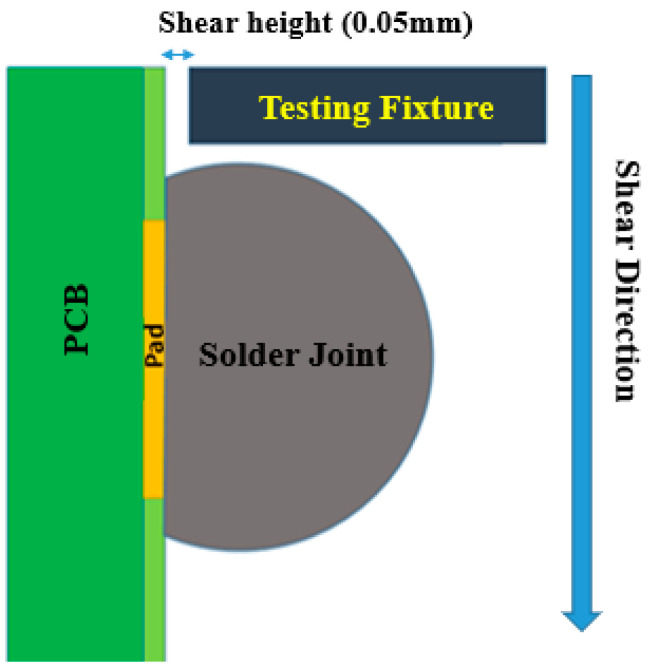
Shear testing schematic.

Ball shear testing was utilized to perform the shear strength test. Although high shear strength alone is not sufficient to ensure the reasonable reliability of solder joints, low shear strength indicates weak solder joints. In our work, an Instron 5948 Micro-Mechanical testing system was used to perform shear strength tests on the aforementioned solder joints. The testing system had a customized fixture. Using a preloaded ball screw drive system, the system achieved an axial displacement resolution of 20 nm. The shear force was measured with a universal 50 N load cell. The shear testing process followed the JESD22-B117 standard for shear testing. The test schematic in shown in Figure 3. On the back side of the PCB, strong glue was applied evenly, and the PCB was glued to a metal workpiece. The shear direction was constantly applied from top to bottom until the solder joint was completely sheared off the Cu pad. According to previous studies, shear strength is affected by two parameters: shear stain rate and shear height. As a result, the shear strain rate and height were chosen to be 0.05 mm and 0.01 s^−1^, respectively. Furthermore, the test was applied to each solder joint at room temperature. The Instron system was used to draw the shear stress–shear strain curves for the specimens aged at 150 °C. The curves were analyzed to determine other mechanical properties. For specimens aged at 100 °C, the ultimate tensile strength was estimated. The peak value of the shear force divided by the cross-sectional area of the solder joint attachment to the pad was used to calculate the ultimate shear strength. 

## 3. Result and Discussion

In this section, results are presented and discussed in terms of microstructure and mechanical properties.

### 3.1. Effect of Aging on Microstructure

**Figure 4:**
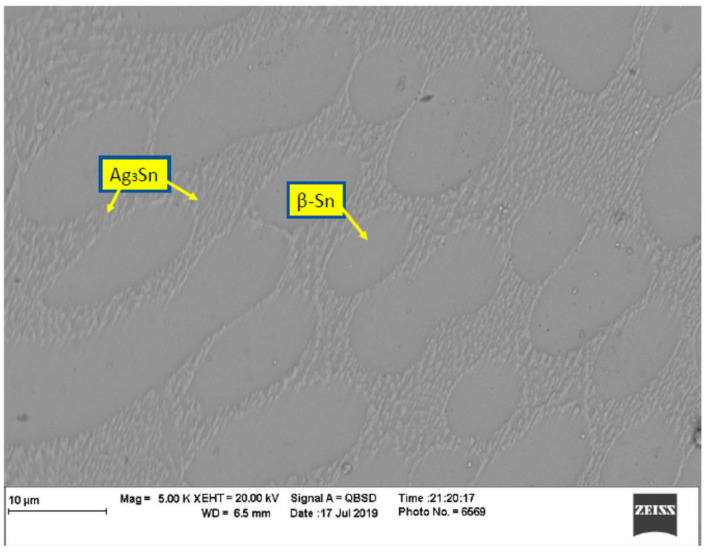
Microstructure of SAC305 as prepared (no aging).

**Figure 5:**
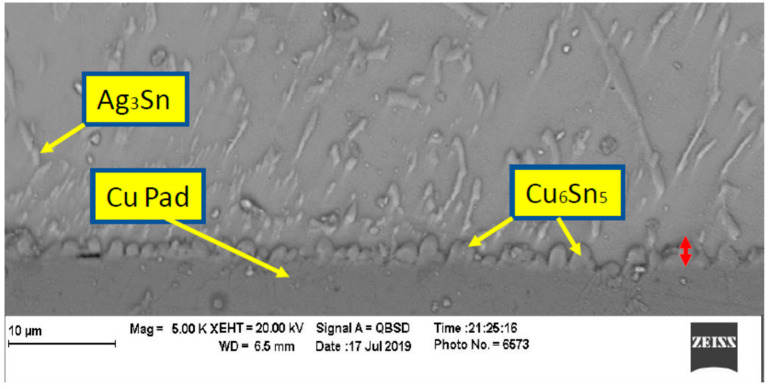
Microstructure of SAC305 fused to a copper pad as prepared (no aging).

**Figure 6:**
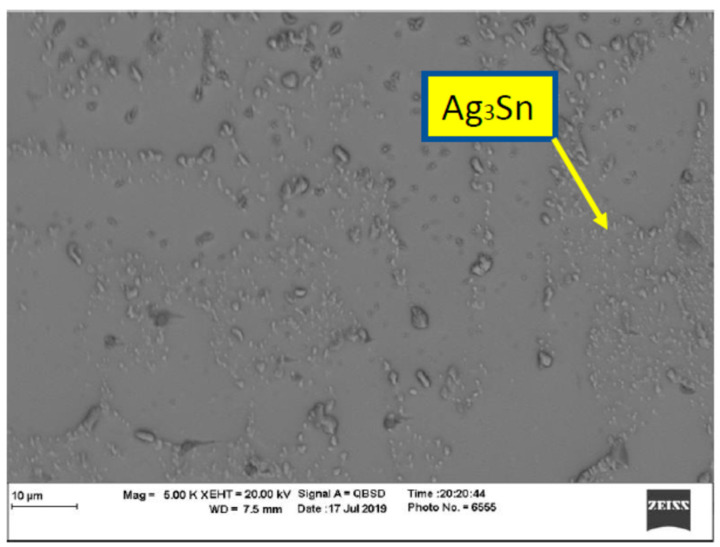
Microstructure of SAC305 thermally aged at 150 °C for 100 h.

**Figure 7:**
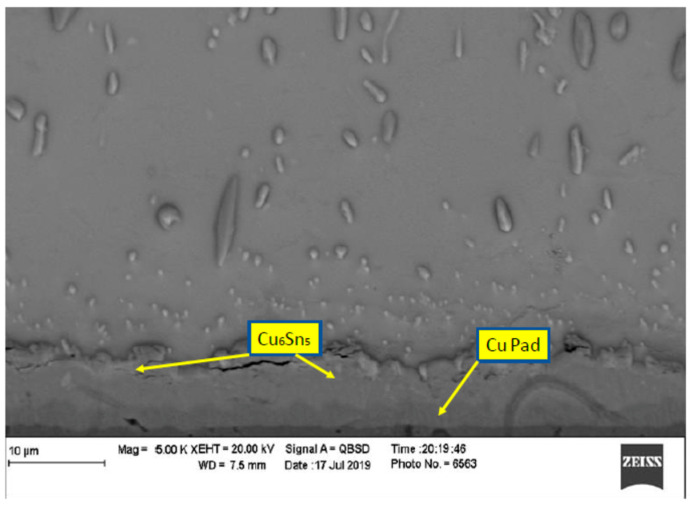
Microstructure of SAC305 fused to a copper pad then thermally aged at 150 °C for 100 h.

**Figure 8:**
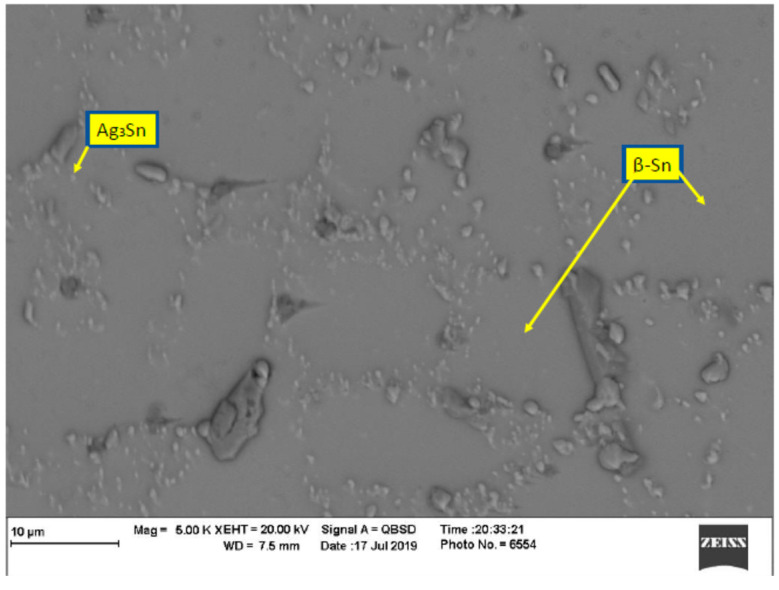
Microstructure of SAC305 thermally aged at 150 °C for 1000 h.

**Figure 9:**
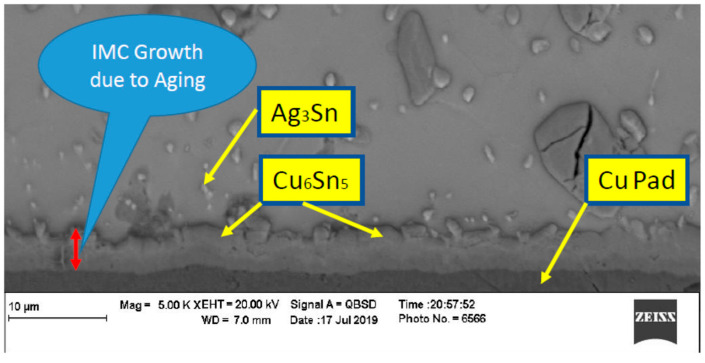
Microstructure of SAC305 fused to a copper pad and then thermally aged at 150 °C for 1000 h.

**Figure 10:**
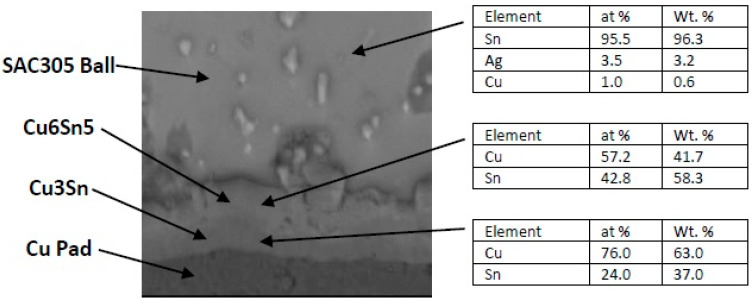
EDAX analysis at select locations on the SAC305/copper interface.

In order to evaluate the thermal aging effects on microstructure, many scanning electronic microscope (SEM) images were taken. The images were organized, filtered, and analyzed. Figure 4 shows the microstructure of SAC305 as prepared, and it is clear that two phases existed, Ag3Sn and β-Sn. There were large spots of β-Sn phase that were completely free of the Ag3Sn. The approximate shape of each spot was oval, and their long diameters typically ranged from 10–20 µm. Because the solder joint shearing experience depends on the diffusion strength between the solder joint and the copper pad, it is important to study the microstructure of this region. Figure 5 shows the area where the joint connected between the copper pad and SAC 305 solder. In this figure, two layers of Cu6Sn5 and Cu3Sn are shown formed over the copper pad. Cu6Sn5 and Cu3Sn are intermetallic compounds (IMCs). After applying thermal aging at a temperature of 150 °C for a period of 100 h, many changes were observed. Figure 6 shows that Ag3Sn was more distributed, and the regular oval spots almost disappeared. However, small regions of β-Sn can still be observed. In Figure 7, more Cu6Sn5 developed and grew to form a large layer adjacent to the copper pad. As the thermal aging duration increased to 1000 h, the previous differences continued to expand. Ag3Sn was further distributed, as shown in Figure 8. Furthermore, the layer of Cu6Sn5 grew additionally, as shown in Figure 9. An interphase crack could form due to material shrinkage during solidification, as shown in Figure 7. Moreover, cracks could form on the phases formed on the surface for the same reason, as shown in Figure 9. Figure 10 shows the energy-dispersive X-ray spectroscopy analysis of the SAC305/copper interface

The IMC thickness was measured after 0, 100, and 1000 h of thermal aging at 150 °C, as shown in Table 2. A thickness of 2 μm was observed without any exposure to thermal aging. This was due to the interaction between copper and molten SAC305. However, the thickness increased with the application of thermal aging, reaching about 6 μm with low variance as shown by images analysis. At 1000 h of aging, the variance in the IMC became really large, and the thickness varied from 6 to 10 μm. Generally, IMC seemingly grows according to a parabolic law. This result was confirmed by a previous study [22]. Although the study in [22] was conducted on a different solder geometry, flat solder on a copper pad, the results are close to those we obtained.

### 3.2. Effect of Thermal Aging on the Mechanical Properties of SAC 305

**Figure 11:**
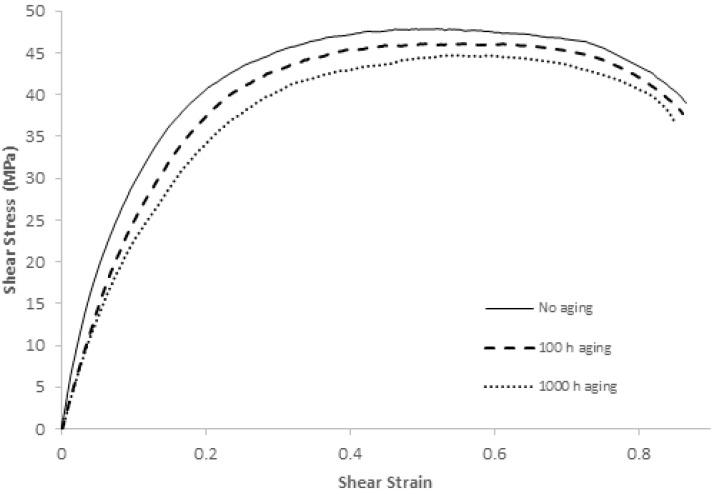
Shear stress–shear strain diagram for samples thermally aged at 150 °C.

As mentioned above, five out of 10 boards were subjected to thermal aging at 150 °C for 0, 2, 10, 100, and 1000 h to help evaluate the mechanical properties of SAC 305 after thermal aging. A shear stress–shear stain diagram was constructed, as shown in Figure 11. In order to minimize error, 10 random solder joints from each board were tested, and the average curve was constructed. The first look at this figure provides a few observations: (1) ultimate shear strength declined with thermal aging, (2) the modulus of elasticity decreased with thermal aging, as shown in Table 3, and (3) toughness decreased with aging. After an analysis of this curve for different aging times, three mechanical properties were extracted, as shown in Table 4: the ultimate shear strength (USS), the total energy exerted to shear off the solder joint, and the ultimate energy to shear off the solder joint. 

Shear stress expresses the force applied to the cross-sectional area of the material, which is parallel to the force applied to the element; shear stress differs from normal stress. It is possible to imagine the effect of shear stress on different materials, such as a saw that cuts a piece of iron. In the current case, the shear stress was applied to a solder joint. Ultimate shear strength is the maximum force divided by the cross-sectional area required to perform the shearing of the solder joint. With longer thermal aging, USS decreases. In order to assess the percentage decrease in USS with increased thermal aging, the drop percentage was calculated by dividing the difference between the current USS and original USS (i.e., 48.58 MPa) by the original USS. The drop was rapid initially and then tapered off. For example, the USS at an aging time of 10 h was 47.24 MPa, down from 48.58 MPa at an aging time of 0 h. This drop is equivalent to 1.34 MPa. However, the decrease in USS between aging times of 100 h and 10 h was 1.13 MPa (i.e., USS of 46 MPa at an aging time of 100 h subtracted from USS of 47.24 MPa at an aging time of 10 h). In other words, the USS decrease between the aging times of 10 and 100 was less than the USS decrease between the aging times of 0 and 10. The same phenomenon can be observed if we take the aging times of 0, 100, and 1000 h and make the same comparison. The USS decrease between the aging times of 100 and 1000 was less than the USS decrease between the aging times of 0 and 100. The drop percentage in USS is presented in column 5 of Table 4. The reflow temperature and time during ball preparation affect the USS, especially if no aging occurs. For example, we applied the reflow process at 245 °C for 45 s, while ref. [22] applied the reflow process at 250 °C for 1 min, and they obtained slightly lower results ours.

The ability to absorb energy without shearing a solder joint under the application of shear stress is called shear toughness. The shear toughness can be derived from the shear stress curve, specifically the area under the curve. The toughness obviously decreased with aging time. Considering the known volume of a solder joint, the total energy (TE) exerted to shear off the solder joint is presented in the third column of Table 4. A very sharp drop in TE was noted in the first 2 h of thermal aging; then, the drop became very slow. The sixth column of Table 4 represents the percentage drop in TE.

Table 1 shows the extracted parameters, namely, the ultimate shear strength, total energy exerted to shear off the solder joint, ultimate energy to shear off the solder joint, percentage change in ultimate shear strength with aging hours, percentage change in total energy required to shear off the solder joint, and percentage change in ultimate shear strength to shear off the solder joint. The last mechanical property is the ultimate energy (UE) required to shear off the solder joint, as presented in the second column of Table 4. This is the amount of energy required to reach USS and then make the failure (i.e., shearing the solder joint) possible. On the stress–shear stain curve, the UE is the area under the curve until the USS point multiplied by the volume of the solder joint. The UE decreases from the beginning of the aging time to a point of inflection, where it begins to increase. The maximum observed UE was 30.97 µJ at an aging time of 10 h. The exact aging time was not estimated at the point of inflection, but it should be between 2 to 100 h. However, approximating this value requires a great deal of effort. It is expected that the reason for the increase and then decrease in UE is that the strain at USS decreased and then increased with aging time. The percentage drop in UE is presented in the seventh column of Table 4. The negative sign means that UE was greater than it was initially. 

**Figure 12:**
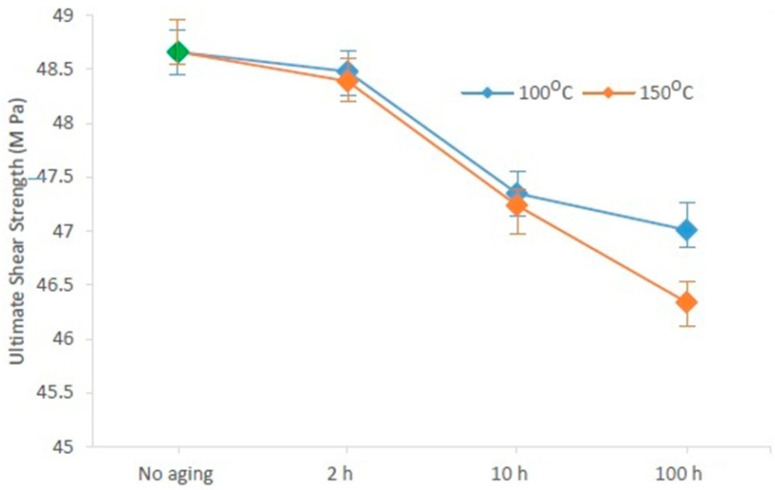
Comparison of USS thermally aged at 100 and 150 °C.

For the boards aged at 100 °C, USS was estimated for 10 solder joints, and the average was considered. Figure 12 shows the effect of aging temperature on USS. It is clear that the drop in USS was higher at higher temperatures. 

## 4. Conclusions

The impact of thermal aging time and temperature on the microstructure and mechanical properties of 96.5Sn–3.0Ag–0.5Cu (SAC305) was investigated. After experimenting, several notes and conclusions could be extracted. Two phases, Ag3Sn and β-Sn inside the lattice, and a third phase, Cu6Sn5 over the copper pad, were formed as SAC305 was produced. With thermal aging, the Ag3Sn phase was more distributed, and the Cu6Sn5 phase formed a thicker layer over the copper pad. Ultimate shear strength declined with thermal aging, the modulus of elasticity increased with thermal aging, and toughness decreased with aging. Furthermore, USS decreased with aging time. However, the decrease was rapid at the beginning and then slowed down. Total energy decreased with aging time. However, a very sharp drop in TE was noted in the first 2 h of thermal aging; then, the drop became very slow. Ultimate energy decreased from the beginning of the aging time to the point of inflection, where it began to increase. Lastly, the drop in USS was clearly higher at higher temperatures.

## Figures and Tables

**Table 1:** Combinations of thermal aging time and temperature.

	0 h	2 h	10 h	100 h	1000 h
150 °C	1 board	1 board	1 board	1 board	1 board
100 °C	1 board	1 board	1 board	1 board	1 board

**Table 2:** IMC thickness at various thermal aging temperatures.

Thickness (µm)	Aging Time (h)
2	0
6	100
6–10	1000

**Table 3:** Modules of elasticity vs. thermal aging time.

Modulus of Elasticity (MPa)	Aging Time
476	0
309	100
278	1000

**Table 4:** Extracted mechanical properties of aged and nonaged SAC 305.

Aging Time	USS (MPa)	Total Energy (µJ)	Ultimate Energy	Drop % USS	Increasing % TE	Increasing % UE
(h)			(µJ)			
0	48.58	39.5	23.49	-	-	-
2	48.4	53.25	27.44	0.38%	41.65%	16.86%
10	47.24	55.92	30.97	2.75%	41.57%	31.86%
100	46.34	56.87	28.68	4.60%	44.00%	22.10%
1000	44.2	58.37	22.79	9.01%	47.78%	−2.97%

## Data Availability

The data presented in this study are available upon request from the corresponding author.
